# Contamination of hybrid hernia meshes compared to bioresorbable Phasix™ Mesh in a rabbit subcutaneous implant inoculation model

**DOI:** 10.1016/j.amsu.2019.08.004

**Published:** 2019-08-13

**Authors:** Spencer P. Lake, Nathaniel F.N. Stoikes, Amit Badhwar, Corey R. Deeken

**Affiliations:** a Department of Mechanical Engineering & Materials Science, Washington University in St. Louis, St. Louis, MO, USA; b Department of Surgery, University of Tennessee Health Science Center, Memphis, TN, USA; c C.R. Bard/Davol. Inc., Warwick, RI, USA; d Covalent Bio, LLC, St. Louis, MO, USA

**Keywords:** Hernia mesh, Hybrid material, Infection, Bacterial colonization, Methicillin-resistant *Staphylococcus aureus* (MRSA), Rabbit model

## Abstract

**Background:**

Hybrid hernia meshes combine biological tissue-derived extracellular matrix with permanent or resorbable synthetic. The objective of this study was to evaluate hybrid meshes (Gore® Synecor, Zenapro™, Ovitex™ 1S Reinforced Bioscaffold Permanent, and Ovitex™ 1S Reinforced Bioscaffold Resorbable) compared to non-hybrid, bioresorbable synthetic mesh (Phasix™ Mesh) in a rabbit bacterial inoculation model.

**Materials and methods:**

Subcutaneous pockets were bilaterally created in male, New Zealand White rabbits (n = 25). Circular meshes (3.8 cm diameter) were implanted and inoculated with 1 × 10^6^ colony forming units (CFU) of clinically-isolated methicillin-resistant *Staphylococcus aureus* (MRSA). A given animal received a single mesh type. Seven days post-inoculation, animals were euthanized and white material and microbial colonization were assessed by abscess scoring and CFU quantification, respectively. Non-parametric Kruskal-Wallis with Dunn's post-hoc tests compared results for different meshes.

**Results:**

Phasix™ Mesh and Synecor exhibited significantly lower abscess scores than Zenapro™, Ovitex™ 1S Permanent, and Ovitex™ 1S Resorbable (*p* < 0.05). All pocket swabs for Zenapro™ and Ovitex™ meshes were positive for MRSA (100%), with 20% of Synecor and 0% Phasix™ Mesh. Microbial colonization was significantly lower for Phasix™ Mesh (0 CFU) relative to Zenapro™ (6.73 × 10^7^ CFU (median)), Ovitex™ 1S Permanent (7.87 × 10^7^ CFU) and Ovitex™ 1S Resorbable (1.45 × 10^8^ CFU), and for Synecor (0 CFU) relative to both Ovitex™ meshes. Phasix™ Mesh was the only device with no detectable abscess or microbial colonization.

**Conclusion:**

Phasix™ Mesh demonstrated no detectable abscess or microbial colonization at 7-days post-implantation and inoculation, in contrast with four hybrid meshes, which all demonstrated colonization in a rabbit bacterial inoculation model.

## Highlights

• Phasix™ Mesh exhibited no detectable abscess or microbial colonization.
• 100% of Zenapro™ and Ovitex™ pocket swabs were positive for MRSA.
• 20% of Synecor pocket swabs were positive for MRSA.
• Phasix™ Mesh exhibited significantly lower abscess scores than Zenapro and Ovitex.
• Synecor exhibited significantly lower abscess scores than Zenapro and Ovitex.

## 1 Introduction

Hernias represent a common clinical problem globally, often requiring surgical repair. The use of mesh has led to improved success rates and reduced hernia recurrence [1,2]. In addition to permanent synthetic materials and biologically-derived products, hybrid hernia repair meshes have been described recently. Hybrid meshes combine synthetic and biological layers, thereby offering benefits of both types of materials [3]. The synthetic layer, comprised of permanent or resorbable polymer fibers, offers mechanical strength to supplement the biological layer, comprised of an extracellular matrix (ECM) scaffold. The ECM is thought to promote tissue ingrowth, limit inflammation, and resist bacterial colonization. These composites offer exciting potential implants that could overcome shortcomings of traditional mesh. However, the performance of hybrid meshes warrants additional study.

A few studies have evaluated hybrid meshes using preclinical animal models. One study examined a poly-lactic-co glycolic acid (PLGA) collagen sponge hybrid compared to a PLGA mesh in a rat model [4], while another study compared a polypropylene mesh combined with porcine small intestinal submucosa (Zenapro™) to bare polypropylene in rats and rabbits [5]. Both studies found hybrid meshes to have promising results regarding regenerative and remodeling attributes and more collagen production compared to controls. In addition to these preclinical studies, a few early clinical investigations of hybrid meshes have been performed. One relevant paper reported results following repair of 63 patients with ventral/incisional hernias using the Zenapro™ hybrid mesh; recurrence rates after 12 months were 6.8%, but no histopathologic data were provided [6]. Another study examined 16 male patients with sports hernias up to 4 months following surgical repair with Zenapro™ [7]. All participants in this preliminary study completed postoperative therapy and returned to sport activity. However, there was relatively little follow-up assessment, which was only short-term, so definitive conclusions are not possible. Another type of hybrid mesh that has been clinically evaluated is Ovitex™ 1S Reinforced Bioscaffold Permanent (TELA Bio, Malvern, PA) [8]. This study evaluated 31 patients following inguinal hernia repair by a single surgeon and found no hernia recurrences, complications, surgical site infections, or chronic postoperative inguinal pain with a short mean follow-up of 12.6 months (range: 3–18 months) [8]. While these early clinical studies have produced favorable results, the properties of hybrid hernia meshes remain poorly understood. In particular, it is not clear how well these composite materials are able to leverage the strengths of the individual synthetic and biologically-derived layers. A more detailed investigation is required to determine their efficacy. The objective of this study was to evaluate an unstudied aspect of hybrid meshes: performance in the presence of contamination. Specifically, the performance of several commercially-available hybrid meshes (Gore® Synecor, Zenapro™, Ovitex™ 1S Reinforced Bioscaffold Permanent, and Ovitex™ 1S Reinforced Bioscaffold Resorbable) was compared to a non-hybrid, bioresorbable synthetic mesh (Phasix™ Mesh) that has previously shown favorable properties.

## 2 Methods

### 2.1 Materials

**Fig. 1:**
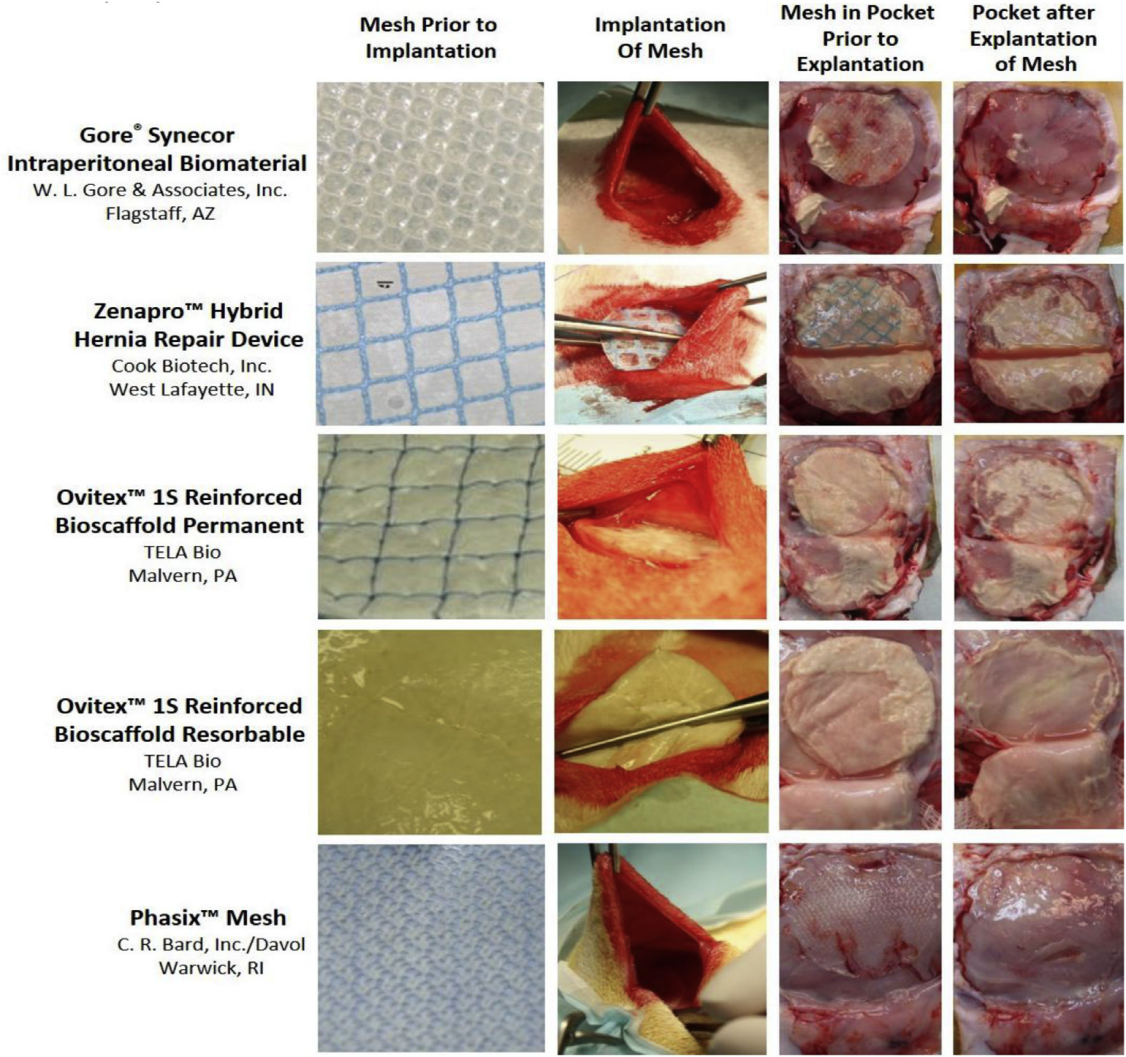
Representative macroscopic photographs of each mesh type prior to implantation (first column), during implantation of mesh into subcutaneous pocket (second column), 7 days after implantation/inoculation with MRSA just prior to explantation of mesh (third column), and after explantation of mesh (fourth column). Note: Each mesh was a 3.8 cm diameter disk prior to implantation.

This study evaluated four hybrid hernia meshes (Fig. 1; first column), including [1]: Gore® Synecor Intraperitoneal Biomaterial (W. L. Gore & Associates, Inc., Flagstaff, AZ), referred to herein as Synecor, composed of a layer of monofilament polytetrafluoroethylene (PTFE) fibers between a nonporous, bioabsorbable poly(glycolide:trimethylene carbonate) copolymer (PGA:TMC) film on the visceral side and a porous, bioabsorbable PGA:TMC web on the parietal side [2,9] Zenapro™ Hybrid Hernia Repair Device (Cook Biotech, Inc., West Lafayette, IN), referred to herein as Zenapro™, composed of a polypropylene mesh between six layers of porcine small intestinal submucosa (SIS) on the visceral side and two layers of porcine SIS on the parietal side [3]; Ovitex™ 1S Reinforced Bioscaffold Permanent (TELA Bio, Malvern, PA), referred to herein as Ovitex™ 1S Permanent, composed of four layers of ovine-derived ECM stitched together using a grid pattern of monofilament polypropylene with an additional two layers of ovine ECM stitched onto one side [10]; and [4] Ovitex™ 1S Reinforced Bioscaffold Resorbable (TELA Bio, Malvern, PA), referred to herein as Ovitex™ 1S Resorbable, composed of four layers of ovine-derived ECM stitched together using a grid pattern of polyglycolic acid (PGA) with an additional two layers of ovine ECM stitched onto one side [11]. Hybrid meshes were compared to a non-hybrid, fully resorbable, biologically-derived mesh (Fig. 1; first column): Phasix™ Mesh (C. R. Bard, Inc./Davol, Warwick, RI), which is a knitted mesh comprised of monofilament fibers of naturally occurring poly-4-hydroxybutyrate (P4HB). Phasix™ Mesh was selected for comparison due to favorable outcomes in previous preclinical studies, particularly in the presence of bacteria [12].

### 2.2 Animal model

This study used an established rabbit model of bacterial inoculation [[13], [14], [15]] with IACUC approval at WuXiAppTec, Inc. (St. Paul, MN) and in accordance with the ARRIVE guidelines [16]. All animals were treated in accordance with the *Guide for the Care and Use of Laboratory* Animals. A total of twenty-five (n = 25) male, New Zealand White rabbits (3.0–3.7 kg; ~16–17 weeks old) were utilized (n = 5/group). (IACUC Protocol #16–549A).

### 2.3 Surgical procedure

Animals were anesthetized using inhalational isoflurane (2.5–5% initially; 0.5–5% throughout procedure). The dorsal area was prepared using sterile technique. Subcutaneous pockets, which are commonly used to evaluate microbial colonization of devices [[13], [14], [15]], were bilaterally created in the dorsal lumbar region of each rabbit. A 2.5–4.5 cm midline incision was created and dissected to expose the paravertebral muscle. The fascial membrane was split and a pocket was created towards the caudal aspect on each side, parallel to the midline, for 7–12 cm. A separate incision was created in each pocket to enable catheter insertion. The tube from a Vacutainer® blood collection set (BD, Franklin Lakes, NJ) was pulled into the pocket and secured to create a bacterial injection catheter. A single, sterile, circular implant (3.8-cm-diameter disk) was inserted into each pocket (Fig. 1; second column), followed by closure of the pocket. A single mesh type was implanted in both pockets of a given animal, with n = 5 animals (n = 10 devices) per mesh group. Pockets were then inoculated with 1 mL containing 1 × 10^6^ colony forming units (CFU) of clinically-isolated methicillin-resistant *Staphylococcus aureus* (MRSA) via the catheter, followed by 1 mL saline flush. The inoculum dose was based on previous studies [12,14,15] and was intended to produce a non-lethal, but viable bacterial infection. The MRSA inoculum was prepared prior to surgery as described previously and solutions were diluted to 1 × 10^6^ CFU [13]. All rabbits were monitored postoperatively and observed twice daily to assess general health and welfare.

### 2.4 Bacterial analysis

Animals were euthanized 7-days post-inoculation via intravenous injection of 150 mg/kg of sodium pentobarbital. At sacrifice, implanted materials from all sites were surgically exposed and gross observations of all implant sites were documented and photographed (Fig. 1; third column). As described previously [[12], [13], [14], [15]], abscess score, pocket swabs positive for MRSA, and bacterial colonization were assessed. The implant site and device were inspected for evidence of white or off-white material indicative of abscess formation. Sites were scored for macroscopic abscess formation as none (0), mild [1], moderate [2], or marked [3]. Implants from each animal were then aseptically explanted and processed for quantitative assessment of the number of viable bacteria remaining on the device and subcutaneous tissue surrounding each implant. Each device was extracted and placed in saline with 0.5% Tween-80. Solutions were serially diluted (10^−1^, 10^−2^, 10^−3^), plated on Trypticase™ Soy Agar (TSA) plates, and cultured at 37 °C for 72 h. Additionally, each implant pocket was photographed (Fig. 1; fourth column) and wiped with sterile swabs, which were then streaked onto TSA and incubated at 37 °C. After 72 h, all plates were examined for microbial growth and colonies were counted (i.e., CFUs). Pocket swabs were considered positive if one or more MRSA colonies were identified.

### 2.5 Statistical analysis

Abscess scores and microbial colonization data were compared across different mesh types using non-parametric Kruskal-Wallis with Dunn's post-test. Statistically significant results were determined for *p* < 0.05. Results from pocket swabs were evaluated qualitatively, with data presented as percentage of positive swabs.

## 3 Results

### 3.1 Gross observations

Two animals implanted with Zenapro™ were humanely euthanized two-days post-operative due to clinical presentation associated with possible sepsis. Thus, subsequent analysis comprised three animals (n = 6 mesh specimens) for Zenapro™ and five animals (n = 10 mesh specimens) for the other groups. Representative photographs after animal sacrifice show implantation sites just prior to (Fig. 1; third column), and immediately following (Fig. 1; fourth column), mesh explantation. All four types of hybrid mesh showed visual evidence of abscess formation, indicative of an inflammatory response and/or bacterial colonization of the implanted biomaterial. Synecor was less severely impacted compared to other hybrids (Zenapro™, OviTex™ 1S Permanent, and OviTex™ Resorbable), while the subcutaneous pockets containing Phasix™ Mesh showed no evidence of abscess formation in any of the animals.

### 3.2 Abscess scoring

**Fig. 2:**
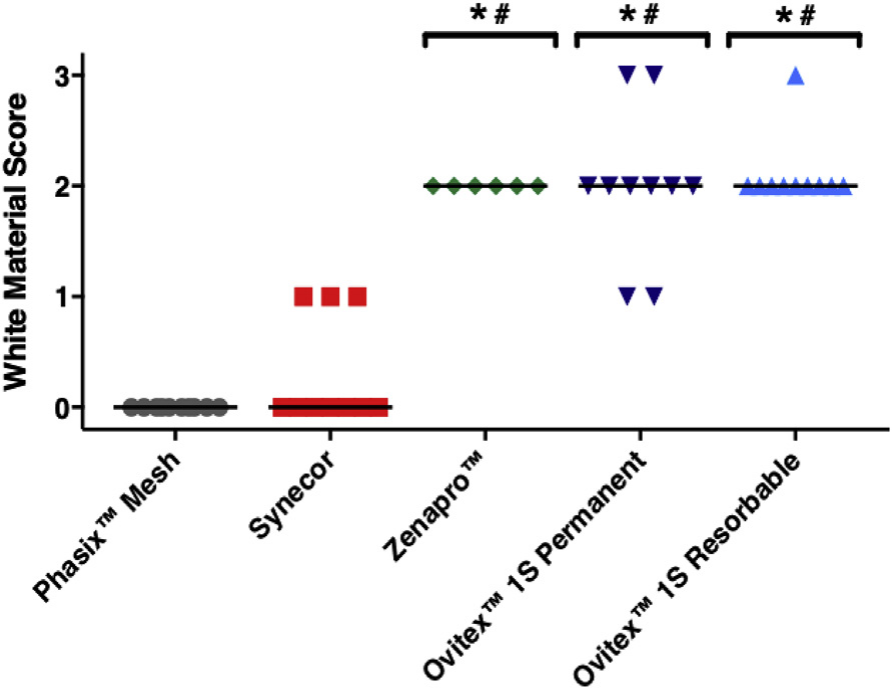
Semi-quantitative abscess analysis demonstrates significantly lower white material scores for Synecor and Phasix™ Mesh compared to Zenapro™, Ovitex™ 1S Permanent, Ovitex™ 1S Resorbable (*p < 0.05 vs. Phasix™ Mesh; #p < 0.05 vs. Synecor; all values are shown with lines representing median values by group.

Phasix™ Mesh and Synecor both exhibited significantly lower abscess scores than Zenapro™, OviTex™ 1S Permanent, and OviTex™ Resorbable (*p* < 0.05) with median values of 0 (Fig. 2). All Phasix™ Mesh devices scored 0; while almost all of the Synecor scores scored 0. Grouped white material scores were not significantly different between Phasix™ Mesh and Synecor (*p* > 0.05). In addition, no significant differences were observed amongst Zenapro™, OviTex™ 1S Permanent and OviTex™ 1S Resorbable, all of which exhibited median white material scores of 2 (i.e., moderate abscess).

### 3.3 Pocket swabs

**Fig. 3:**
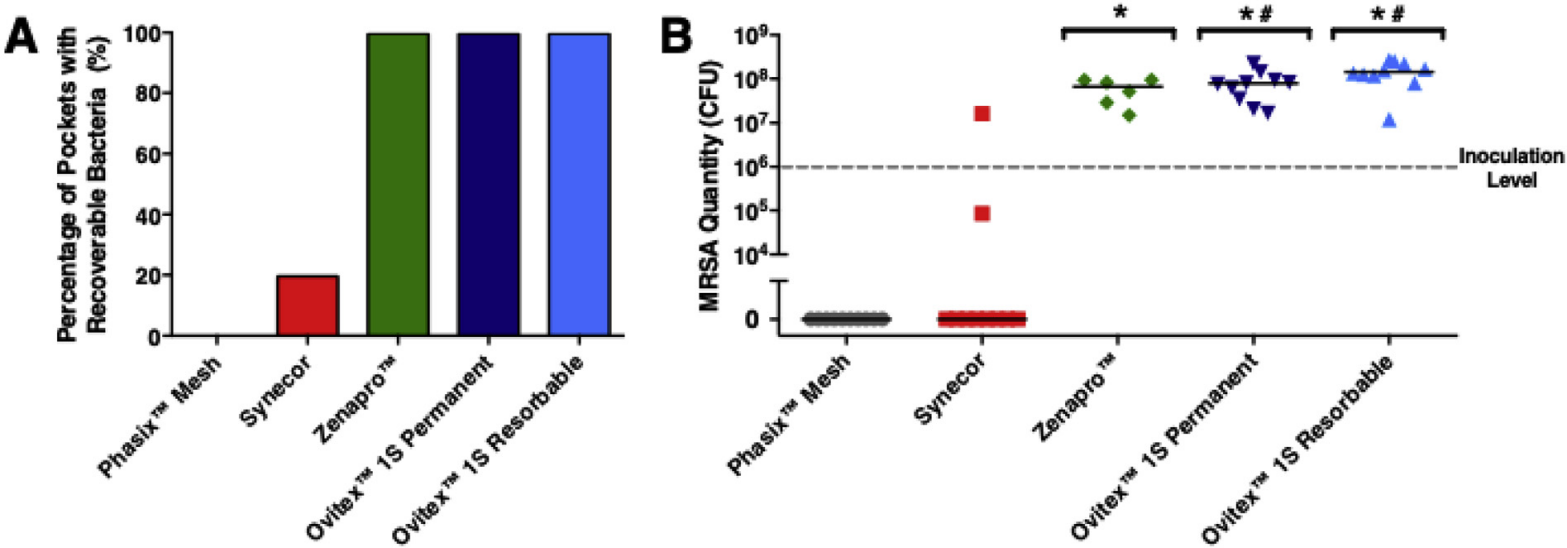
(A) Percentage of pockets for each mesh type that contained recoverable bacteria and (B) measured quantity of MRSA extracted from pockets containing meshes (reported in colony forming units or CFU) demonstrate favorable bacterial clearance for Synecor and superior bacterial clearance for Phasix™ Mesh compared to other hybrid meshes tested (*p < 0.05 vs. Phasix™ Mesh; #p < 0.05 vs. Synecor; all values are shown with lines representing median values by group).

At 7-days post inoculation, Phasix™ Mesh had 0% (0/10) pockets that contained recoverable bacteria, while Synecor exhibited 20% (2/10) of pockets that were positive for MRSA (Fig. 3A; Table 1). In contrast, 100% of subcutaneous pockets contained bacteria when implanted with Zenapro™ (6/6), OviTex™ 1S Permanent (10/10), and OviTex™ 1S Resorbable (10/10) meshes.

**Table 1:** Evaluation of microbial colonization of different types of implanted meshes, reported as colony forming units (CFU) of MRSA. Note: the initial inoculation level was 10^6^ CFU of MRSA. (n = 10 for all groups except Zenapro™ with n = 6).

Phasix™ Mesh	Synecor	Zenapro™	Ovitex™ 1S Permanent	Ovitex™ 1S Resorbable
0	1.64E+07	9.38E+07	1.68E+07	2.53E+08
0	8.58E+04	1.49E+07	2.07E+07	1.68E+08
0	0	5.18E+07	9.33E+07	1.19E+08
0	0	2.88E+07	8.28E+07	2.69E+08
0	0	8.28E+07	3.50E+07	2.18E+08
0	0	9.50E+07	6.23E+07	1.35E+08
0	0	--	2.24E+08	1.28E+08
0	0	--	7.70E+07	1.56E+08
0	0	--	8.05E+07	1.21E+07
0	0	--	1.46E+08	8.00E+07

### 3.4 Bacterial colonization

For each of the different mesh groups, microbial colonization was assessed via CFU quantification (Fig. 3B; Table 1). None of the Phasix™ Mesh implant sites demonstrated bacterial colonization; the median CFU score for Phasix™ Mesh (0 CFU) was significantly smaller (p < 0.05) than Zenapro™ (6.72 × 10^7^ CFU), OviTex™ 1S Permanent (7.87 × 10^7^ CFU), and OviTex™ Resorbable (1.45 × 10^8^ CFU). Synecor had only two samples that exhibited non-zero bacterial colonization (8.58 × 10^4^ CFU and 1.64 × 10^7^ CFU), with significantly lower median CFU scores (0 CFU) than OviTex™ 1S Permanent and OviTex™ Resorbable (*p* < 0.05). Bacterial colonization was not significantly different between Synecor and Phasix™ Mesh or between Synecor and Zenapro™ (*p* > 0.05). Finally, CFU values were not significantly different between Zenapro™ and either of the OviTex™ 1S meshes (Permanent or Resorbable) (*p* > 0.05). All viable bacteria recovered from implant sites were identified as *Staphylococcus aureus.*

## 4 Discussion

In this study, a rabbit bacterial inoculation model was utilized to evaluate how several hybrid meshes and a comparison bioresorbable mesh responded following direct inoculation with clinically-isolated MRSA. One of the hybrid meshes, Synecor, showed a favorable overall response with low abscess scores and little recoverable bacteria 7-days following implantation/inoculation. In contrast, the other three hybrid meshes evaluated (Zenapro™, OviTex™ 1S Permanent, and OviTex™ 1S Resorbable) exhibited significantly greater bacterial colonization as assessed via gross observation and quantification of collected MRSA. In fact, Zenapro™, OviTex™ 1S Permanent, and OviTex™ 1S Resorbable showed colonization 10–100 times greater (10^7^-10^8^) than the initial inoculation level (1 × 10^6^).

A potential advantage of hybrid meshes is to leverage the strengths of all materials that comprise the mesh. The synthetic material is included to provide mechanical integrity and strength, while the biological layers are intended to support a positive host response, including the promotion of tissue ingrowth, limitation of inflammation, and a theoretical heightened resistance to bacterial colonization. Contrary to this ideal, the results of the current study showed significant bacterial colonization for three of the four evaluated hybrid meshes (Zenapro™, OviTex™ 1S Permanent, and OviTex™ 1S Resorbable).

The non-hybrid Phasix™ Mesh demonstrated abscess scores of zero, negative pocket swabs, and zero cases of positive bacterial colonization in contrast with the four hybrid meshes. This favorable response may be related to the macroporous, monofilament fiber structure of Phasix™ Mesh. Previous studies have shown that increased surface area of mesh materials (e.g., multifilament) enables greater bacterial adherence to implanted biomaterials compared to mesh designs with less surface area (e.g., monofilament) [12,[17], [18], [19], [20]]. Therefore, a porous structure may provide less material upon which bacteria can adhere and colonize compared to tightly woven or mat-like mesh materials found in many hybrid meshes. Thus, mesh morphology should be considered during mesh selection, along with material type. Another potential contributing factor may be release of antimicrobial peptides (AMPs), as recent evidence suggests that implantation of Phasix™ Mesh induces greater upregulation and release of AMPs than other resorbable meshes [21]. The bactericidal effect of AMPs may limit bacterial survival and colonization on the implanted biomaterial. Consistent with this hypothesis, a previous preclinical study showed that Phasix™ Mesh exhibited a better response to bacterial inoculation than a fully resorbable synthetic mesh [12]. Of note, the previous preclinical study [12] used an inoculation level that was 100x greater than the present study (i.e., 1 × 10^8^ CFU MRSA), demonstrating favorable performance of Phasix™ Mesh at even higher bacterial levels than what was evaluated in the present study. It should be noted that most, but not all, of the Phasix™ Mesh devices in the previous study exhibited 0 CFU (median; interquartile range: 0–3.75 × 10^2^ CFU) with 22% of pocket swabs positive for MRSA.

Other studies have considered the performance of biologically-derived meshes using various bacterial inoculations. For example, one study examined six commercially-available biologically-derived meshes using both *in vitro* (agar plate culture) and *in vivo* (dorsal subcutaneous rabbit model) analyses [14]. Seven days following inoculation of MRSA (5 × 10^7^ CFU) or *Escherichia coli* (1 × 10^6^ CFU), significant bacterial colonization was observed for all meshes except for the antibiotic-coated, non-crosslinked porcine acellular dermal matrix (XenMatrix™ AB, C. R. Bard/Davol, Inc., Warwick, RI). Although direct comparison to the present study is not possible due to study differences, the results suggest a limited ability of uncoated biologically-derived materials to inhibit bacterial colonization and reduce inflammation, similar to what was observed for several of the hybrid meshes in the present study.

This study used methicillin-resistant *Staphylococcus aureus* (MRSA) bacteria, the most common cause of surgical site infection [22,23], to evaluate the mesh response. Many mesh infections are polymicrobial, and other types of bacteria could have been evaluated. However, MRSA was chosen for analysis in this study because it represents a challenging and common infection. MRSA has also been used in several previous studies that similarly evaluated bacterial colonization of biomaterial implants [12,14,15], thus it is well accepted as a representative bacterium.

## 5 Conclusion

This study evaluated the performance of several hybrid meshes and a non-hybrid bioresorbable synthetic mesh in the presence of MRSA inoculation in an animal model. Synecor showed lower abscess scores and decreased CFU values compared to the other three hybrid meshes. The non-hybrid comparison mesh (Phasix™ Mesh) demonstrated no detectable abscess or microbial colonization at 7-days post-implantation and inoculation, in contrast with four hybrid meshes, which all demonstrated colonization in a rabbit bacterial inoculation model. Further study is needed to evaluate other properties of hybrid meshes compared to synthetic or biologic meshes to establish their role in hernia repair.

## Ethical approval

Not applicable to this preclinical study.

## Sources of funding

This study was funded by Davol, Inc. (Warwick, RI), a subsidiary of C. R. Bard, Inc. (Franklin Lakes, NJ). Bard and Davol have joined BD (Franklin Lakes, NJ).

Study conception and design: Badhwar.

Acquisition of data: Badhwar.

Analysis and interpretation of data: Lake, Stoikes, Badhwar, Deeken.

Drafting the article or revising critically for intellectual content: Lake, Stoikes, Badhwar, Deeken.

Final approval of the submitted version: Lake, Stoikes, Badhwar, Deeken.

## Author contribution

Study conception and design: Badhwar.

Acquisition of data: Badhwar.

Analysis and interpretation of data: Lake, Stoikes, Badhwar, Deeken.

Drafting the article or revising critically for intellectual content: Lake, Stoikes, Badhwar, Deeken.

Final approval of the submitted version: Lake, Stoikes, Badhwar, Deeken.

## Conflicts of interest

Dr. Stoikes is a consultant for, and Dr. Badhwar is an employee of, C. R. Bard, Inc. Dr. Lake is a consultant for, and Dr. Deeken is the owner of, Covalent Bio, LLC, which received funding from C. R. Bard, Inc. for this project, as well as other, unrelated projects.

## Research registration number

Not applicable to this preclinical study.

## Guarantor

Amit Badhwar, PhD.

## Provenance and peer review

Not commissioned, externally peer reviewed.
